# Emerging Trends in Diagnostic Radiology: Integrating Advanced Imaging Modalities for Early Detection, Clinical Monitoring, and Prognostic Evaluation of Multisystem Internal Medicine Disorders

**DOI:** 10.7759/cureus.101488

**Published:** 2026-01-13

**Authors:** Shubham Gupta, Pankaj Kaira, Shagufa Pathan, Krishna Chidrawar, Krupa Rajeshbhai Gondaliya, Sandeep Kaur Toor

**Affiliations:** 1 Department of Radiodiagnosis, Government Medical College (GMC) Jammu, Jammu, IND; 2 Department of Radiodiagnosis, Shri Ram Murti Smarak Institute of Medical Sciences, Bareilly, IND; 3 Department of Otorhinolaryngology, Dharmsinh Desai University, Dr. N.D. Desai Faculty of Medical Science and Research, Nadiad, IND; 4 Department of Radiodiagnosis, Maharashtra University of Health Sciences, Nashik, IND; 5 Department of Emergency, Government Medical College and New Civil Hospital, Surat, IND; 6 Department of Radiodiagnosis, Punjab Institute of Liver and Biliary Sciences, Mohali, IND

**Keywords:** artificial intelligence, diagnostic radiology, imaging biomarkers, multimodality imaging, precision medicine

## Abstract

Diagnostic radiology has progressed from basic X-ray imaging to a highly advanced, multimodality discipline that integrates high-resolution structural techniques, functional and molecular imaging, and computational analytics. This review synthesizes emerging trends in diagnostic radiology with a particular focus on their relevance to multisystem internal medicine disorders. The historical evolution of imaging is outlined, from early radiographs to computed tomography (CT), magnetic resonance imaging (MRI), and hybrid platforms such as positron emission tomography-computed tomography (PET-CT) and positron emission tomography-magnetic resonance imaging (PET-MRI). The transition toward precision medicine is highlighted through the use of quantitative imaging biomarkers and radiomics, which enable detailed phenotyping, early disease detection, and individualized treatment planning. Key applications across cardiovascular, respiratory, endocrine, autoimmune, and hematologic disorders are discussed, demonstrating how multimodality imaging improves diagnostic accuracy, guides therapy, and supports longitudinal monitoring. Challenges related to resource availability, cost-effectiveness, radiation safety, data standardization, and ethical considerations in artificial intelligence (AI) are also addressed. Looking forward, innovations such as theranostics, automated image analysis, cloud-based tele-radiology, and multidisciplinary integration are expected to further strengthen radiology’s role in personalized, value-based care. These advancements underscore the central importance of diagnostic imaging in modern internal medicine and the need for continued research and equitable global implementation.

## Introduction and background

Multisystem internal medicine disorders constitute a substantial global health burden because they involve multiple organ systems simultaneously and often require complex, multidisciplinary diagnostic and therapeutic approaches [1]. Conditions such as systemic autoimmune diseases, cardiovascular-metabolic syndromes, sarcoidosis, and hematologic malignancies frequently present with heterogeneous clinical manifestations, making early and accurate diagnosis challenging [2]. Delays in diagnosis can lead to increased morbidity, higher healthcare costs, and poorer quality of life, underscoring the importance of timely and precise disease detection [3,4].

Diagnostic radiology has long been integral to internal medicine, evolving from conventional plain radiography to advanced multimodal imaging capable of visualizing structural, functional, and molecular disease processes [5]. The advent of high-resolution computed tomography (HRCT), advanced magnetic resonance imaging (MRI) techniques, positron emission tomography-computed tomography (PET-CT), and hybrid imaging platforms has significantly enhanced the ability to comprehensively evaluate multisystem disorders [6-8]. These innovations have facilitated earlier diagnosis, improved disease characterization, and more accurate assessment of disease extent across organ systems.

Related work

Previous studies have demonstrated the clinical value of advanced imaging modalities in specific multisystem conditions. For example, cardiac MRI enables detailed myocardial tissue characterization, allowing early detection of myocarditis and accurate phenotyping of cardiomyopathies [9-11]. Similarly, fluorodeoxyglucose (FDG)-PET-CT provides whole-body metabolic imaging that is particularly useful in systemic inflammatory and neoplastic conditions such as vasculitis, lymphoma, and sarcoidosis [12-14]. These modalities represent a shift from purely anatomical imaging toward functional and quantitative assessment, supporting more precise phenotyping and prognostication [15,16].

More recently, artificial intelligence (AI) and radiomics have emerged as transformative tools in diagnostic radiology [17,18]. By enabling automated feature extraction, risk stratification, and predictive modeling, these technologies have the potential to reduce interobserver variability, standardize reporting, and optimize resource utilization [19,20]. In parallel, the expansion of tele-radiology and mobile imaging platforms has improved access to advanced diagnostic services, particularly in resource-limited settings, contributing to greater equity in healthcare delivery [21-23].

Building on this evolving body of work, the present review synthesizes emerging trends in diagnostic radiology with a focus on their integrated application to multisystem internal medicine disorders. It examines the role of advanced and hybrid imaging modalities, including PET-CT, PET-MRI, functional MRI, HRCT, and advanced ultrasound techniques in early diagnosis, clinical management, and prognostic evaluation. Applications across cardiovascular, respiratory, endocrine, metabolic, autoimmune, and hematologic disorders are discussed within the broader framework of precision medicine and multidisciplinary care.

Methodology

This narrative review was conducted using a structured and systematic approach to ensure a comprehensive, balanced, and clinically relevant synthesis of the available literature on emerging trends in diagnostic radiology. The methodological framework was designed to accommodate the heterogeneity of study designs, imaging modalities, and clinical applications inherent to multisystem internal medicine disorders. Given this heterogeneity, including variability in clinical indications, imaging technologies, and outcome measures, a qualitative narrative synthesis was considered the most appropriate methodological approach, as quantitative pooling or meta-analysis was not feasible or methodologically justified.

Literature Search Strategy

A comprehensive literature search was performed across multiple electronic databases, including PubMed, Scopus, Web of Science, and Google Scholar, to capture a broad spectrum of peer-reviewed research in radiology, internal medicine, and multidisciplinary imaging applications. In addition to original research articles, authoritative clinical guidelines and consensus statements from major professional societies, such as the American College of Radiology (ACR), Radiological Society of North America (RSNA), and European Association of Nuclear Medicine (EANM), were included to ensure alignment with current clinical standards and best practices.

The search strategy employed Boolean operators (AND/OR) and combined relevant keywords with Medical Subject Headings (MeSH) terms related to advanced imaging and multisystem disease. Search terms included, but were not limited to, diagnostic radiology, multimodality imaging, multisystem disorders, PET-CT, PET-MRI, functional MRI, high-resolution CT, artificial intelligence in radiology, and radiomics. The search was restricted to publications from January 2015 to March 2025 to focus on recent technological advances, particularly in hybrid imaging, quantitative biomarkers, and AI-driven analytics. Only English-language articles published in peer-reviewed journals were considered.

Eligibility Criteria

Clear inclusion and exclusion criteria were applied to enhance methodological rigor. Eligible publications included original research studies, systematic reviews, meta-analyses, narrative reviews, and consensus guidelines that addressed the diagnostic, monitoring, or prognostic role of imaging in multisystem internal medicine disorders. Studies limited to isolated case reports, conference abstracts, editorials, letters to the editor, or non-peer-reviewed sources were excluded, as were articles published in languages other than English.

Study Selection and Data Extraction

Study selection followed a two-stage screening process. Initially, titles and abstracts were reviewed to identify potentially relevant publications. Articles meeting preliminary relevance criteria were then subjected to full-text review to confirm eligibility. Key data elements were extracted from selected studies, including study design, patient population, imaging modality, clinical indication, primary outcomes, and relevance to multisystem disease management. Extracted data were organized thematically according to imaging modality and clinical application.

Evidence Synthesis

Given the heterogeneity of imaging technologies, study designs, and clinical endpoints, evidence synthesis was conducted using a qualitative narrative approach. Emphasis was placed on identifying areas of convergence across studies, clinically meaningful trends, strengths and limitations of existing evidence, and gaps in validation or implementation. This integrative synthesis was used to contextualize technological advancements within real-world clinical practice, highlight current best practices, and outline future research priorities in diagnostic radiology for multisystem internal medicine disorders.

## Review

Evolution of diagnostic radiology

Historical Milestones

The discipline of diagnostic radiology has undergone a remarkable transformation since Wilhelm Conrad Röntgen discovered X-rays in 1895, which marked the birth of medical imaging. Initially, radiology relied on plain film radiographs that provided only two-dimensional representations of anatomy. Although ahead of their era, early radiographs had poor contrast of soft tissues and difficulty in distinguishing between overlapping structures [3]. With the advent of fluoroscopy and contrast-enhanced studies at the beginning of the 20th century, diagnostic possibilities were broadened, and the dynamic assessment of the gastrointestinal and vascular systems became possible. Digital imaging technologies emerged in the late 20th century and greatly enhanced the quality of images, efficiency of workflow, and archiving. The replacement of the analog film with digital radiography and computed radiography supported the development of Picture Archiving and Communication Systems (PACS), which allows remote access and connection with the hospital information systems [15]. These developments paved the way to the networked radiology practice that is today data-driven.

Emergence of Multimodality Imaging

The advent of cross-sectional imaging that began with computed tomography (CT) in the 1970s was a paradigm shift since it offered the prospect of tomographic visualization of internal structures at a high spatial resolution. CT quickly became the gold standard in the evaluation of trauma, pulmonary embolism (PE), and staging of cancer [24]. Neuroimaging, musculoskeletal imaging, and cardiac imaging were revolutionized in the 1980s with the introduction of magnetic resonance imaging (MRI), which provided superior soft tissue contrast without ionizing radiation [25].

Another significant breakthrough was the use of hybrid imaging methods, which use a combination of structural and functional data in a single study. PET-CT has become an essential tool in cancer treatment, staging, and monitoring of malignancies, including lymphoma and breast cancer [17,26]. Recently, PET-MRI has had the benefit of reduced radiation dose and improved soft tissue characterization, and is especially useful in neuro-oncology, pediatric imaging, and inflammatory disorders [16,18]. On the same note, single-photon emission computed tomography combined with CT (SPECT-CT) has improved the assessment of bone pathology, cardiac perfusion, and endocrine diseases [20].

Multimodality imaging has allowed clinicians to go beyond an anatomical description of the pathophysiology to a complete examination of the pathophysiology, including morphologic, metabolic, and functional information. Indicatively, non-invasive evaluation of anatomical stenosis and its hemodynamic importance can now be achieved using coronary CT angiography with CT-derived fractional flow reserve (CT-FFR) as opposed to invasive angiography [27]. The whole-body MRI (WB-MRI) is also becoming a popular method of assessing systemic inflammatory disorders and hematologic malignancies, and is a radiation-free technique that can be used to monitor the disease over time [8].

Transition to Precision Medicine

Standardized values considered quantitative imaging biomarkers for PET, such as standardized uptake values (SUVs), and for MRI, such as apparent diffusion coefficient (ADC) values, and volumetric tumor burden and can be used to monitor disease progression and treatment response over time [11]. Radiomics, i.e., high-throughput extraction of quantitative characteristics of medical images, is now an efficient form of tumor phenotyping, prognostication, and therapy selection [12]. One such case is that radiomic signatures have been linked to molecular subtypes in hepatocellular carcinoma and prostate cancer, which can offer the chance to non-invasive precision oncology [10,28].

This is changing through the addition of machine learning and artificial intelligence (AI), which automate the process of image segmentation, lesion detection, and predictive analytics. Smart triage systems may be implemented to prioritize the urgent results, decrease the reporting turnaround time, and help the radiologists in the pattern recognition activities using AI [29]. Moreover, predictive models based on artificial intelligence (AI) are being designed to help predict the disease course and personalize a treatment plan by combining imaging, clinical, and genomic information [21].

The example of the integration of multimodality imaging in the multidisciplinary care pathways testifies to the presence of radiology as one of the centers of precision medicine [30]. Radiology has since become an aggressive player in therapeutic decision-making, whether it be patient selection of patients who will receive targeted biologics or the construction of image-guided treatment and evaluation of therapeutic response [22]. This change highlights the fact that image interpretation has become data interpretation, and thus, radiologists have become an indispensable part of the precision healthcare team.

Advanced imaging technologies

The recent advancement in diagnostic radiology has resulted in a variety of new high-tech imaging modalities, which provide more anatomical, functional, and molecular data on the pathophysiology of disease. These modalities are important in the diagnosis of early and correct staging, therapy, and prognostic monitoring of multisystem internal medicine disorders. Table 1 provides an integrative overview of major imaging modalities, their clinical applications, and representative supporting literature.

**Table 1:** Summary of Advanced Imaging Modalities, Key Clinical Applications, and References AI: artificial intelligence, CT: computed tomography, HRCT: high-resolution computed tomography, ICU: intensive care unit, MRI: magnetic resonance imaging, OR: operating room, PET-CT: positron emission tomography-computed tomography, PET-MRI: positron emission tomography-magnetic resonance imaging, POCUS: point-of-care ultrasound, SPECT-CT: single-photon emission computed tomography combined with CT

Modality	Primary clinical applications	Representative references
HRCT	Evaluation of interstitial lung disease, chronic obstructive pulmonary disease, and pulmonary embolism	[29]
Functional MRI (perfusion/diffusion)	Brain ischemia, traumatic brain injury, tumor grading, and cardiac perfusion assessment	[6,16,25]
PET-CT	Oncologic staging, therapy response assessment, and surveillance for lymphoma and breast cancer	[17,26]
PET-MRI	Pediatric and neuro-oncology imaging, inflammatory disorders, and soft tissue characterization	[16,18]
SPECT-CT	Bone metastasis detection, cardiac perfusion, and endocrine imaging	[20]
Radiomics and AI	Image segmentation, prognostic modeling, predictive analytics, and workflow triage	[11,12,19]
POCUS	Rapid bedside evaluation of thoracic, cardiac, and abdominal conditions	[20]
Portable CT/handheld MRI	Imaging in ICU/OR settings, trauma, and neurocritical care	[31]

High-Resolution and Functional Imaging

High-resolution computed tomography (HRCT) remains the gold standard for the evaluation of interstitial lung disease (ILD), offering detailed visualization of lung parenchyma and enabling early identification of fibrotic patterns and disease progression [29]. Functional MRI techniques, including perfusion and diffusion imaging, have transformed neuroimaging by allowing the assessment of cerebral blood flow, ischemic penumbra, and microstructural integrity in traumatic brain injury and neurodegenerative disorders [16,25]. In cardiovascular medicine, cardiac MRI with perfusion and late gadolinium enhancement has become indispensable for characterizing myocarditis, cardiomyopathy, and myocardial viability [6,27]. Diffusion-weighted imaging and diffusion tensor imaging further expand oncologic and neurologic applications by enabling tumor grading, therapy response assessment, and white matter tractography [10].

Molecular and Hybrid Imaging

Molecular imaging modalities provide a window into cellular and biochemical processes, enabling early detection of pathology before structural changes become apparent. PET-CT is widely employed in oncology for staging, therapy response, and surveillance of malignancies such as lymphoma, breast cancer, and neuroblastoma [17,26]. PET-MRI offers similar metabolic insights with the added advantage of superior soft tissue contrast and reduced radiation exposure, making it a preferred modality in pediatric oncology, neuro-oncology, and inflammatory disorders [16,18]. Likewise, SPECT-CT has enhanced the evaluation of bone metastases, cardiac perfusion, and endocrine pathologies by combining functional and anatomical information, improving diagnostic specificity [20].

Artificial Intelligence and Radiomics

Artificial intelligence (AI) and radiomics are reshaping radiology practice by enabling quantitative feature extraction, automated pattern recognition, and predictive modeling. Machine learning algorithms can segment organs, detect lesions, and classify disease phenotypes with high reproducibility, supporting radiologists in high-volume workflows [19]. Radiomics enables the conversion of standard imaging data into high-dimensional quantitative features that can predict tumor biology, treatment response, and patient outcomes [11]. These tools also hold potential for building multiparametric prognostic models when combined with clinical and genomic data [12]. AI-driven triage systems further streamline workflow by flagging urgent cases, reducing turnaround time, and enhancing diagnostic efficiency [21].

Despite these efficiency gains, the clinical impact of AI and radiomics remains uneven across practice settings. Many proposed algorithms are derived from retrospective or single-center datasets, and their generalizability across scanners, institutions, and patient populations remains insufficiently validated. Moreover, improvements in workflow efficiency do not consistently translate into demonstrated improvements in patient-centered outcomes, highlighting the need for prospective, multicenter studies and standardized validation frameworks.

Point-of-Care and Portable Imaging

Bedside and portable imaging technologies are critical for critically ill patients and resource-limited settings. Point-of-care ultrasound (POCUS) is now widely used for rapid assessment of pleural effusion, pericardial tamponade, pneumothorax, and abdominal free fluid, making it a mainstay in emergency and intensive care medicine [30]. Advances in portable CT scanners and emerging handheld MRI systems allow imaging in operating rooms and ICUs without the need for patient transport, reducing risks and improving the timeliness of diagnosis [31].

Applications in multisystem internal medicine disorders

Cardiovascular Disorders

Cardiovascular imaging has undergone a technological renaissance, particularly with the advent of non-invasive coronary imaging. Coronary CT angiography (CCTA) offers high spatial resolution for the detection of coronary artery disease and is now widely used in both symptomatic patients and risk-stratified populations [32]. The addition of CT-derived fractional flow reserve (CT-FFR) allows for physiological assessment of lesion-specific ischemia, reducing unnecessary invasive angiography and guiding revascularization decisions [27].

Cardiac MRI has now been the gold standard of tissue characterization of the myocardium. The differentiation between ischemic and non-ischemic cardiomyopathies, and T1 and T2 mapping makes it possible to identify the diffuse myocardial fibrosis, edema, and infiltration, respectively [6]. They play an important role in the phenotyping of myocarditis and amyloidosis, and the risk selection of arrhythmia and heart failure. Moreover, cardiac MRI is also becoming a growing trend and is becoming more popular as a tool to assess cardiotoxicity of chemotherapy, and it is a sensitive method of longitudinal surveillance [9].

Respiratory Disorders

High-resolution computed tomography (HRCT) has revolutionized the interstitial lung diseases (ILDs) of pulmonary medicine. It allows seeing fine ground-glass opacities, reticulations, honeycombing, and traction bronchiectasis, and can be used to distinguish idiopathic pulmonary fibrosis and other types of ILD [29]. Phenotyping of chronic obstructive pulmonary disease (COPD) is another area in which the HRCT is a very important factor, as the measurements of emphysema and air trapping are applied to perform intervention. The CT perfusion and dual-energy CT have expanded the diagnostic tools of pulmonary embolism (PE). These techniques provide not only visualization of emboli directly but also functional assessment of perfusion defects, which increases the certainty of diagnosing and risk stratification [32]. The ability to measure the vascular obstruction and parenchymal perfusion simultaneously in the examination can provide timely anticoagulation and monitoring of response to treatment.

Endocrine and Metabolic Disorders

Diagnostic radiology is used in the treatment of endocrine and metabolic diseases. Diabetic microvascular complications, such as neuropathy and nephropathy, can be imaged using high-resolution MRI, and the structural alteration of the peripheral nerves and kidneys can be detected at an early stage before irreversible damage is caused [33]. Similarly, musculoskeletal MRI can identify early diabetic Charcot neuroarthropathy, which can be offloaded and avoided before the development of deformities.

Molecular imaging with PET has transformed the diagnosis and localization of neuroendocrine tumors (NETs). ^68^Ga-DOTATATE PET-CT has superior sensitivity compared to conventional imaging and is now the gold standard for NET staging, restaging, and peptide receptor radionuclide therapy (PRRT) planning [28]. Additionally, ^18^F-FDG PET and ^11^C-methionine PET are increasingly utilized in parathyroid imaging for the localization of adenomas in challenging cases, guiding minimally invasive parathyroidectomy [17].

Autoimmune, Rheumatologic, and Hematologic Disorders

Whole-body MRI (WB-MRI) has emerged as a powerful tool for assessing autoimmune and rheumatologic diseases in the entire body. It is not radiative, and it can be applied to test various organ systems, and that is why it is appropriate in the case of systemic lupus erythematosus, systemic sclerosis, and idiopathic inflammatory myopathies [34]. WB-MRI is highly sensitive for the detection of myositis, synovitis, and osteitis, and early diagnosis and monitoring of therapy [31].

PET imaging, especially FDG-PET-CT, has a significant role in systemic vasculitides, as it reveals vessel wall inflammation even prior to the structural changes manifesting themselves [8]. It is also popular in staging and response evaluation of hematologic malignancies, including lymphoma and multiple myeloma [18]. The added advantage of hybrid PET-MRI is that it has less radiation exposure and better soft tissue resolution, thus becoming more popular in younger patients and those who need serial follow-up.

Across these clinical domains, although multimodality imaging improves diagnostic confidence and disease characterization, evidence demonstrating direct impact on long-term outcomes, cost-effectiveness, and treatment modification remains heterogeneous. This highlights the need for disease-specific prospective studies and standardized endpoints to support broader clinical adoption.

**Figure 1:**
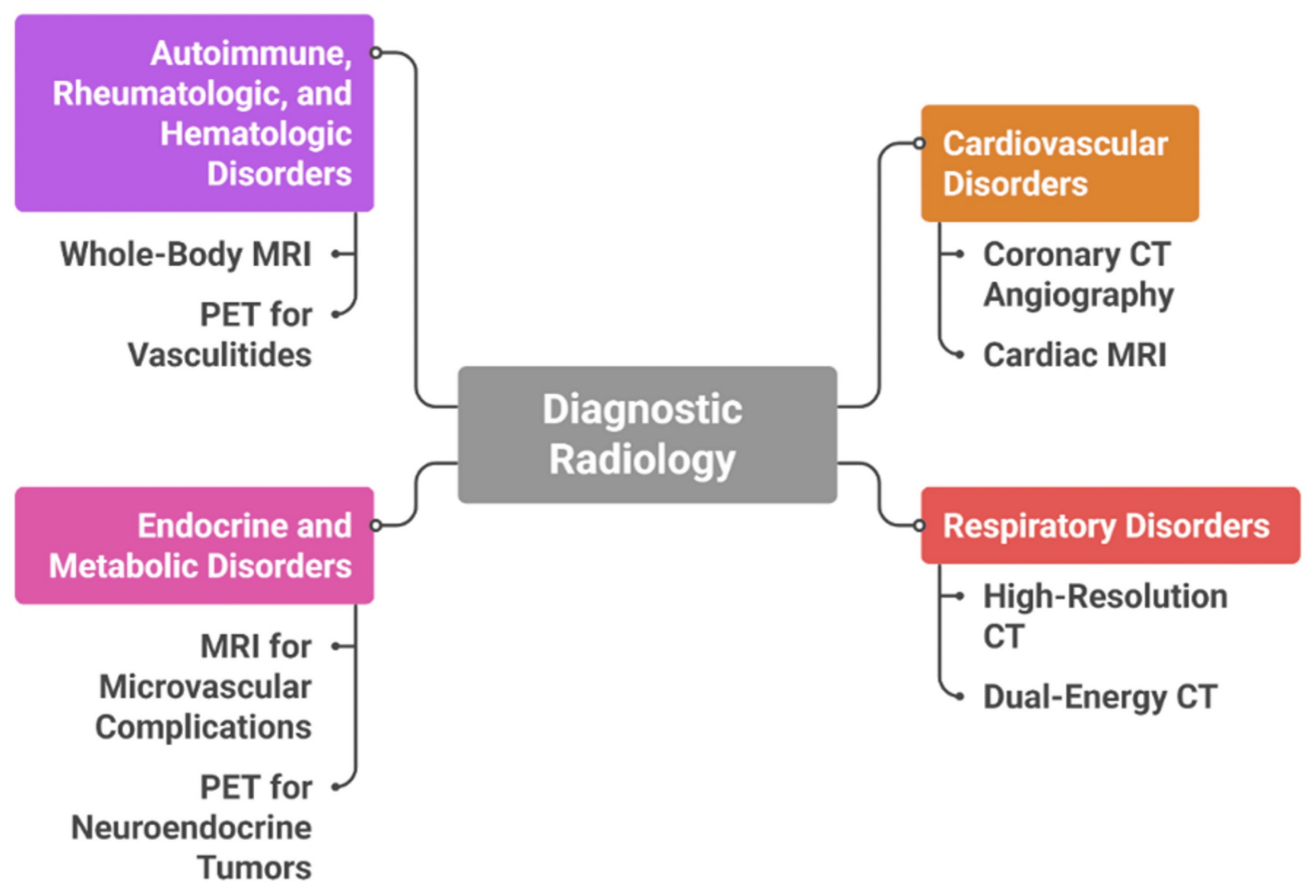
Applications of Diagnostic Radiology in Major Clinical Disorders CT: computed tomography, MRI: magnetic resonance imaging, PET: positron emission tomography Created by the authors

Collectively, these applications illustrate how multimodality imaging enhances diagnostic accuracy, guides therapeutic interventions, and facilitates longitudinal monitoring across diverse internal medicine specialties. By integrating anatomical, functional, and molecular data, radiology serves as a central pillar in the multidisciplinary management of complex multisystem disorders. As shown in Figure 1, diagnostic radiology integrates specialized imaging modalities across multiple clinical disorders, illustrating both shared diagnostic pathways and areas where modality-specific evidence continues to evolve.

Clinical monitoring and prognostic evaluation

Imaging Biomarkers for Disease Activity

Quantitative imaging biomarkers have become essential tools for assessing disease activity across multiple organ systems. In oncology, volumetric tumor burden, metabolic tumor volume, and standardized uptake values (SUVs) derived from FDG-PET-CT are used to monitor treatment response and detect early relapse [17]. These parameters are particularly relevant in lymphoma, breast cancer, and multiple myeloma, where metabolic response precedes anatomical change [18]. Likewise, apparent diffusion coefficient (ADC) values measured by MRI are early indicators of tumor cellularity and chemotherapy/radiotherapy response, which can be used to adaptively treat [10].

Late gadolinium enhancement (LGE) extent and extracellular volume (ECV) fraction on cardiac MRI are imaging biomarkers that are associated with the burden of myocardial fibrosis and potent predictors of arrhythmic risk and adverse outcomes in cardiovascular disorders [6,9]. Whole-body MRI has been used in rheumatology to measure synovial and bone marrow edema scores, which are used as a substitute for disease activity in spondyloarthropathies and rheumatoid arthritis [34]. Dual-energy CT, in a similar manner, is capable of quantifying urate crystal deposition in gout, which is a quantitative biomarker to monitor the efficacy of therapy [30]. Additional functional parameters include perfusion indices, myocardial strain, and fractional flow reserve obtained with CT (CT-FFR), which help in assessing the disease activity. As an illustration, serial CT-FFR measurements have the potential to show progression or regression of flow-limiting coronary lesions and can be used to intensify or de-escalate medical therapy [27].

Despite their growing clinical use, many imaging biomarkers lack universally accepted thresholds and remain subject to inter-scanner, inter-vendor, and inter-observer variability, which can limit reproducibility and comparability across institutions. Furthermore, most validation studies are retrospective, underscoring the need for prospective, outcome-driven trials to establish their definitive role in clinical decision-making.

Longitudinal Monitoring

Chronic conditions that require long-term follow-ups include interstitial lung disease, vasculitis, inflammatory bowel disease, and systemic autoimmune conditions. Imaging has been the key in determining the right intervals of monitoring and evaluation of therapeutic response with time. HRCT is applied both at baseline and at set intervals in ILD to identify progression, response to antifibrotic treatment, and lung transplantation [29].

Serial CT, MRI, or PET imaging is an important response assessment criterion used in oncology (RECIST 1.1 and Lugano Classification) to assess the success of treatment and the necessity to change the regimen or start second-line therapy. FDG-PET-CT enables monitoring of metabolic response following a single or two cycles of chemotherapy and provides the possibility of treatment modification in non-responders [8].

In chronic cardiovascular disease, serial cardiac MRI can monitor remodeling in cardiomyopathy and track regression of hypertrophy or fibrosis with targeted therapies. Similarly, MR angiography and PET imaging allow surveillance of aortic and large vessel vasculitides, identifying subclinical inflammation that may warrant immunosuppression escalation [24].

However, optimal imaging intervals, cost-effectiveness, and the impact of repeated imaging on long-term patient outcomes remain incompletely defined, particularly in chronic non-oncologic diseases. Evidence-based surveillance strategies tailored to disease activity and risk profiles are still evolving.

Prognostic Models

The integration of imaging biomarkers with clinical and genomic data is ushering in an era of personalized prognostic modeling. Radiomics, in particular, enables the extraction of hundreds of quantitative imaging features that can be correlated with molecular signatures and outcomes [11]. For instance, radiomic signatures in hepatocellular carcinoma have been linked to microvascular invasion and overall survival, guiding selection for transplantation or loco-regional therapy [10].

Artificial intelligence (AI)-driven predictive analytics are being developed to forecast disease progression, relapse risk, and treatment toxicity. Machine learning models trained on multimodal datasets, combining imaging, laboratory values, and genomics, have demonstrated superior accuracy compared to conventional risk scores in conditions such as glioma, prostate cancer, and heart failure.

Importantly, these prognostic models are increasingly being embedded into clinical workflows, allowing dynamic risk assessment and individualized therapy. For example, in lymphoma, interim PET response and radiomic features can predict progression-free survival, enabling risk-adapted intensification or de-escalation of chemotherapy.

Nevertheless, widespread clinical adoption of AI-driven prognostic models is constrained by limited external validation, a lack of transparency in algorithmic decision-making, and uncertainty regarding clinical accountability, highlighting the need for standardized evaluation frameworks and regulatory oversight. As summarized in Table 2, these imaging biomarkers and prognostic models underpin personalized care by enabling longitudinal disease tracking and risk stratification, while also highlighting areas where validation and clinical integration remain ongoing challenges.

**Table 2:** Imaging Biomarkers, Monitoring Strategies, and Prognostic Models Across Multisystem Disorders ADC: apparent diffusion coefficient, AI: artificial intelligence, CT-FFR: CT-derived fractional flow reserve, DECT: dual-energy computed tomography, DLCO: diffusion capacity of the lungs for carbon monoxide, ECV: extracellular volume, HRCT: high-resolution computed tomography, ILD: interstitial lung disease, LGE: late gadolinium enhancement, PET: positron emission tomography, SUVmax: maximum standardized uptake value, WB-MRI: whole-body magnetic resonance imaging

Domain	Key imaging biomarkers/monitoring tools	Prognostic utility	References
Oncology	Tumor volume, SUVmax, ADC values, radiomics features	Predicts treatment response, relapse risk, and survival	[12,16]
Cardiovascular	LGE extent, ECV fraction, CT-FFR	Stratifies arrhythmia risk and guides revascularization decisions	[15,19]
Respiratory	Serial HRCT fibrosis score, DLCO correlation	Monitors ILD progression and determines timing for transplant	[18]
Rheumatologic/metabolic	WB-MRI synovitis score, DECT urate crystal volume	Guides therapy escalation and predicts flare risk	[17,30]
Vasculitis/hematology	PET metabolic activity, vessel wall uptake	Identifies active inflammation and predicts relapse	[25,26]
AI/radiomics models	High-dimensional feature sets integrated with genomics	Builds predictive models for survival and therapy response	[11,31]

Challenges and limitations

Resource and Cost Constraints

The availability of state-of-the-art imaging modalities is often restricted in low- and middle-income countries (LMICs), where access to PET-CT, PET-MRI, and advanced MRI sequences is limited by high acquisition costs, maintenance requirements, and shortages of trained personnel [35]. Even in well-resourced healthcare systems, the rapid expansion of imaging indications and increased reliance on advanced technologies have contributed to rising healthcare expenditure, raising concerns regarding overutilization, sustainability, and equitable access [21].

Cost-effectiveness analyses are essential to justify the adoption of expensive imaging modalities, particularly in screening and longitudinal follow-up settings. For example, whole-body MRI for multiple myeloma surveillance may reduce cumulative radiation exposure but incurs higher direct costs compared with conventional skeletal surveys. Similarly, while AI-based workflow automation and decision-support tools can improve reporting efficiency and throughput, their implementation requires substantial upfront investment in software, infrastructure, validation studies, and workforce training. Reimbursement policies often lag behind technological innovation, delaying widespread clinical adoption of modalities such as PET-MRI and AI-enabled diagnostic systems [36].

Safety and Radiation Concerns

Radiation exposure remains a critical consideration, particularly in chronic diseases requiring repeated imaging. Although modern CT systems employ dose-reduction strategies such as iterative reconstruction and automated exposure control, cumulative radiation exposure from serial examinations continues to pose potential long-term risks. Pediatric patients and young adults with chronic illnesses are especially vulnerable, necessitating strict justification of imaging indications and adherence to the As Low As Reasonably Achievable (ALARA) principle [37].

Hybrid imaging modalities such as PET-CT further compound radiation dose concerns, whereas PET-MRI offers a partial mitigation strategy by eliminating CT-based attenuation correction. Ongoing efforts to optimize radiation safety include weight-based radiotracer dosing, low-dose CT protocols, and advanced reconstruction algorithms that preserve image quality while minimizing exposure [38]. Accreditation programs and regulatory bodies, including the American College of Radiology (ACR), have established quality assurance frameworks, dose benchmarks, and continuing education requirements to standardize safety practices across imaging centers.

Standardization and Interoperability

A major barrier to translating imaging innovations into routine clinical practice is the lack of standardized reporting frameworks and data interoperability. Despite the availability of structured reporting systems such as the Liver Imaging Reporting and Data System (LI-RADS) and Prostate Imaging Reporting and Data System (PI-RADS), adoption remains inconsistent, resulting in variability in reporting quality and clinical interpretation [39]. This challenge is further amplified in AI-driven imaging, where heterogeneity in acquisition protocols, scanner vendors, and annotation standards limits reproducibility and external validation of algorithms [40].

Efforts to address these limitations include the use of Digital Imaging and Communications in Medicine (DICOM) standards, structured reporting templates, and open-source imaging repositories. However, achieving true interoperability will require coordinated collaboration among equipment manufacturers, healthcare institutions, regulatory agencies, and professional societies to support data harmonization and multicenter AI development [12,41].

Ethical, Security, and Privacy Considerations in AI

The increasing integration of artificial intelligence into medical imaging workflows has improved diagnostic efficiency, reduced reporting turnaround time, and enhanced image interpretation. However, these benefits have also raised significant ethical, legal, and societal concerns affecting clinicians, patients, healthcare systems, regulators, and the public. A key challenge lies in the validation and generalizability of AI models, many of which are trained on single-center or demographically homogeneous datasets, raising concerns about algorithmic bias and inequitable performance across populations.

Transparency and explainability remain critical issues, as clinicians must understand AI-generated outputs to maintain trust, accountability, and appropriate clinical oversight. From a security perspective, the use of large imaging datasets and cloud-based platforms introduces vulnerabilities related to data breaches, cyberattacks, and unauthorized access. Compliance with data protection regulations such as the Health Insurance Portability and Accountability Act (HIPAA) and the General Data Protection Regulation (GDPR) is essential, particularly as tele-radiology and federated learning models expand across institutional and national boundaries.

**Figure 2:**
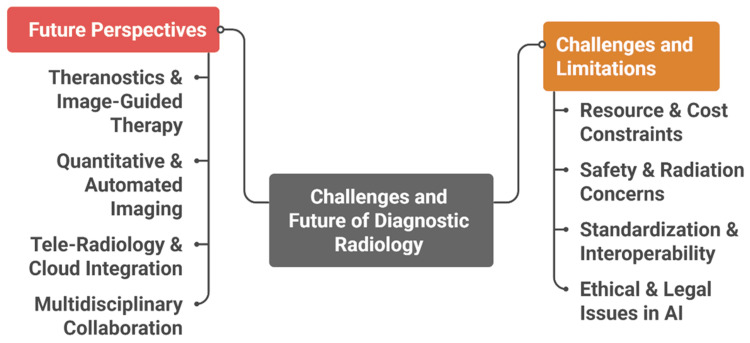
Challenges, Limitations, and Future Perspectives in Diagnostic Radiology AI: artificial intelligence Created by authors

Legal and ethical questions regarding liability, responsibility for diagnostic errors, and informed patient consent remain areas of active debate. Regulatory agencies, including the US Food and Drug Administration (FDA) and the European Commission, are developing frameworks to address these concerns; however, governance models for continuous learning AI systems are still evolving [27]. Figure 2 summarizes key technical, ethical, and operational challenges in diagnostic radiology and outlines potential future directions for responsible implementation.

Future perspectives

Theranostics and Image-Guided Therapy

The concept of theranostics, combining diagnostics with targeted therapy, is gaining traction, particularly in oncology. Molecular imaging is now used not only for detection but also for selecting patients for radionuclide therapies, such as ^177^Lu-DOTATATE for neuroendocrine tumors and ^177^Lu-PSMA for metastatic prostate cancer [42,43]. Image-guided therapy, including MRI-guided focused ultrasound, transarterial radioembolization, and CT-guided ablation, represents another frontier where radiology actively contributes to curative interventions.

Quantitative and Automated Imaging

Quantitative imaging biomarkers are expected to play an increasingly central role in precision medicine. Fully automated pipelines capable of extracting volumetric, textural, and functional parameters from images will facilitate reproducible, high-throughput analyses [44]. The combination of radiomics, radiogenomics, and AI will enable more accurate prognostic modeling and therapy response prediction, moving radiology from a qualitative to a quantitative science [45]. Automation also has the potential to alleviate workforce shortages by performing time-consuming tasks such as segmentation, lesion tracking, and report generation, allowing radiologists to focus on integrative decision-making.

Tele-Radiology and Cloud Integration

Tele-radiology is poised to expand further, leveraging cloud infrastructure to enable global image sharing, remote consultations, and 24/7 coverage [46]. This is particularly impactful for rural and underserved regions where access to subspecialty radiologists is limited. Cloud-based platforms also facilitate large-scale research collaborations, enabling federated learning approaches that train AI models across institutions without sharing raw data, thereby preserving patient privacy [47].

Multidisciplinary Collaboration

The future of radiology lies in deeper integration within multidisciplinary teams. Radiologists are increasingly participating in tumor boards, heart teams, and inflammatory disease boards, contributing to holistic clinical decision-making [48]. This model of collaboration will make sure that imaging results are placed into the larger clinical context and treatment plans are optimized for each patient. These trends will require education and training to focus on data science, informatics, and communication skills to train radiologists to assume leadership positions in precision medicine efforts [49,50]. Altogether, despite the issues of cost, access, safety, standardization, and ethical implementation of AI, the future of diagnostic radiology is likely to be more personalized, more automated, and accessible to everyone worldwide. The future of radiology in today’s internal medicine may and should be the source of improved patient outcomes in patients across the world, through embracing innovation, standardization, and multidisciplinary collaboration.

## Conclusions

The evolution of diagnostic radiology from conventional radiography to advanced multimodal imaging platforms has fundamentally reshaped the practice of internal medicine by enabling earlier diagnosis, refined disease characterization, and improved prognostic assessment across multiple organ systems. The integration of high-resolution imaging, functional MRI, hybrid molecular techniques such as PET-CT and PET-MRI, and AI-driven analytics allows simultaneous evaluation of anatomical, functional, metabolic, and molecular disease signatures. These developments have positioned radiology as a core component of precision medicine, supporting individualized diagnosis, therapy selection, treatment monitoring, and longitudinal risk stratification. Radiomics and artificial intelligence further expand this role by automating image analysis, reducing interobserver variability, and facilitating predictive modeling and real-time clinical decision support.

Despite these advances, significant challenges remain that limit widespread clinical translation. Much of the supporting evidence for advanced imaging biomarkers and AI-based tools remains heterogeneous and predominantly retrospective, with limited prospective validation demonstrating direct impact on patient-centered outcomes. Issues related to standardization, reproducibility across platforms, cost-effectiveness, and equitable access continue to constrain implementation. In parallel, the rapid integration of AI into medical imaging raises critical concerns regarding data privacy, algorithmic transparency, bias, accountability, and ethical governance. Given the multisystem scope of this review, breadth was intentionally prioritized to provide an integrated overview of emerging trends; however, this approach also highlights the need for future disease-specific, outcome-driven studies and standardized evaluation frameworks. Continued progress will depend on rigorous validation research, harmonized reporting standards, secure data infrastructures, and close multidisciplinary collaboration to ensure that diagnostic radiology delivers measurable, value-based improvements in patient care.

## References

[1] Bahadoram S, Davoodi M, Hassanzadeh S, Bahadoram M, Barahman M, Mafakher L (2022). Renal cell carcinoma: an overview of the epidemiology, diagnosis, and treatment. G Ital Nefrol.

[2] Vilaplana Grosso F (2019). Orthopedic diagnostic imaging in exotic pets. Vet Clin North Am Exot Anim Pract.

[3] Mori H, Torii S, Kutyna M, Sakamoto A, Finn AV, Virmani R (2018). Coronary artery calcification and its progression: what does it really mean?. JACC Cardiovasc Imaging.

[4] Cannistraro RJ, Badi M, Eidelman BH, Dickson DW, Middlebrooks EH, Meschia JF (2019). CNS small vessel disease: a clinical review. Neurology.

[5] Ghafoor S, Burger IA, Vargas AH (2019). Multimodality imaging of prostate cancer. J Nucl Med.

[6] Hasan M, Nadkarni N, Rajpurohit N (2024). Advanced cardiac imaging modalities: a brief review for the primary care physician. S D Med.

[7] Elwazir MY, Bois JP, Abou Ezzeddine OF, Chareonthaitawee P (2020). Imaging and quantification of cardiac sarcoidosis. Semin Nucl Med.

[8] Ricci F, Mantini C, Grigoratos C (2019). The multi-modality cardiac imaging approach to cardiac sarcoidosis. Curr Med Imaging Rev.

[9] Bucciarelli-Ducci C, Ajmone-Marsan N, Di Carli M, Nicol E (2022). The year in cardiovascular medicine 2021: imaging. Eur Heart J.

[10] Afyouni S, Zandieh G, Nia IY, Pawlik TM, Kamel IR (2024). State-of-the-art imaging of hepatocellular carcinoma. J Gastrointest Surg.

[11] Maino C, Vernuccio F, Cannella R (2024). Radiomics and liver: where we are and where we are headed?. Eur J Radiol.

[12] Haddadi Avval A, Banerjee S, Zielke J, Kann BH, Mueller S, Rauschecker AM (2025). Applications of artificial intelligence and advanced imaging in pediatric diffuse midline glioma. Neuro Oncol.

[13] Otsuka H (2019). Clinical imaging technology and the diagnosis in patient-centered interdisciplinary care. J Med Invest.

[14] Smith LG, Milliron E, Ho ML, Hu HH, Rusin J, Leonard J, Sribnick EA (2019). Advanced neuroimaging in traumatic brain injury: an overview. Neurosurg Focus.

[15] Farooqi KM, Cooper C, Chelliah A (2019). 3D printing and heart failure: the present and the future. JACC Heart Fail.

[16] Keir G, Roytman M, Mashriqi F, Shahsavarani S, Franceschi AM (2024). Atypical parkinsonian syndromes: structural, functional, and molecular imaging features. AJNR Am J Neuroradiol.

[17] Paydary K, Seraj SM, Zadeh MZ (2019). The evolving role of FDG-PET/CT in the diagnosis, staging, and treatment of breast cancer. Mol Imaging Biol.

[18] Shah SN, Oldan JD (2017). PET/MR imaging of multiple myeloma. Magn Reson Imaging Clin N Am.

[19] Qureshi AY, Stevens RD (2022). Mapping the unconscious brain: insights from advanced neuroimaging. J Clin Neurophysiol.

[20] Erba PA, Bartoli F, Sollini M, Raffaella B, Zanca R, Enrica E, Lazzeri E (2022). Alternative nuclear imaging tools for infection imaging. Curr Cardiol Rep.

[21] Lai J, Wang B, Petrik M, Beziere N, Hammoud DA (2023). Radiotracer development for fungal-specific imaging: past, present, and future. J Infect Dis.

[22] Trabulsi EJ, Rumble RB, Jadvar H (2020). Optimum imaging strategies for advanced prostate cancer: ASCO guideline. J Clin Oncol.

[23] Di Donna A, Masala S, Muto G, Marcia S, Giordano F, Muto M (2024). Metabolic bone diseases: recommendations for interventional radiology. Semin Musculoskelet Radiol.

[24] Carroll BJ, Schermerhorn ML, Manning WJ (2020). Imaging for acute aortic syndromes. Heart.

[25] Smith CP, Laucis A, Harmon S, Mena E, Lindenberg L, Choyke PL, Turkbey B (2019). Novel imaging in detection of metastatic prostate cancer. Curr Oncol Rep.

[26] Swift CC, Eklund MJ, Kraveka JM, Alazraki AL (2018). Updates in diagnosis, management, and treatment of neuroblastoma. Radiographics.

[27] Garg I, Grist TM, Nagpal P (2023). MR angiography for aortic diseases. Magn Reson Imaging Clin N Am.

[28] Oprea-Lager DE, MacLennan S, Bjartell A (2024). European Association of Nuclear Medicine Focus 5: consensus on molecular imaging and theranostics in prostate cancer. Eur Urol.

[29] Liszewski MC, Ciet P, Winant AJ, Lee EY (2022). Lung and large airway imaging: magnetic resonance imaging versus computed tomography. Pediatr Radiol.

[30] Li X, Cokkinos D, Gadani S (2022). Advanced ultrasound techniques in arterial diseases. Int J Cardiovasc Imaging.

[31] Monti R, Martinese A, Bonadonna G (2022). Degenerative and inflammatory musculoskeletal disorders: updates and hot topics in diagnostic and interventional imaging. Eur Rev Med Pharmacol Sci.

[32] Bruno F, Albano D, Agostini A (2023). Imaging of metabolic and overload disorders in tissues and organs. Jpn J Radiol.

[33] Maheshwari S, Gu CN, Caserta MP (2024). Imaging of alcohol-associated liver disease. AJR Am J Roentgenol.

[34] Vyas S, Bhalla AS, Ranjan P, Kumar S, Kumar U, Gupta AK (2016). Rheumatoid arthritis revisited - advanced imaging review. Pol J Radiol.

[35] Cohen-Rosenblum A, Cui Q (2019). Osteonecrosis of the femoral head. Orthop Clin North Am.

[36] Dijkema EJ, Leiner T, Grotenhuis HB (2017). Diagnosis, imaging and clinical management of aortic coarctation. Heart.

[37] Rao HL, Pradhan ZS, Suh MH, Moghimi S, Mansouri K, Weinreb RN (2020). Optical coherence tomography angiography in glaucoma. J Glaucoma.

[38] Arıbal E (2023). Future of breast radiology. Eur J Breast Health.

[39] Park EH, Fritz J (2023). The role of imaging in osteoarthritis. Best Pract Res Clin Rheumatol.

[40] Parakh A, Tirkes T (2020). Advanced imaging techniques for chronic pancreatitis. Abdom Radiol (NY).

[41] Ma KK, Lin S, Mok VC (2019). Neuroimaging in vascular parkinsonism. Curr Neurol Neurosci Rep.

[42] Daniels SP, Fritz J (2023). Acute and chronic elbow disorders: MR imaging-ultrasonography correlation. Magn Reson Imaging Clin N Am.

[43] McGettigan M, Zulfiqar M, Shetty AS (2023). Imaging of vaginal and vulvar malignancy. Radiol Clin North Am.

[44] Moore MM, Gee MS, Iyer RS (2022). ACR Appropriateness Criteria® Crohn disease-child. J Am Coll Radiol.

[45] Jha P, Pōder L, Glanc P (2022). Imaging cancer in pregnancy. Radiographics.

[46] Gilger BC (2017). Advanced imaging of the equine eye. Vet Clin North Am Equine Pract.

[47] Belkin DA, Wysong A (2016). Radiographic imaging for skin cancer. Semin Cutan Med Surg.

[48] Khessib T, Jha P, Davidzon GA, Iagaru A, Shah J (2024). Nuclear medicine and molecular imaging applications in gynecologic malignancies: a comprehensive review. Semin Nucl Med.

[49] Prerna Prerna, Chadha J, Khullar L, Mudgil U, Harjai K (2024). A comprehensive review on the pharmacological prospects of terpinen-4-ol: from nature to medicine and beyond. Fitoterapia.

[50] Chadha J, Ahuja P, Mudgil U, Khullar L, Harjai K (2024). Citral and triclosan synergistically silence quorum sensing and potentiate antivirulence response in Pseudomonas aeruginosa. Arch Microbiol.

